# Successful Management of Limbal Dermoid in Infancy and Childhood: A Case Series

**DOI:** 10.7759/cureus.22835

**Published:** 2022-03-04

**Authors:** Dina M Abdulmannan

**Affiliations:** 1 College of Medicine, Umm Al-Qura University, Makkah, SAU

**Keywords:** management, limbal, infancy, dermoid, childhood

## Abstract

This is a case series study that reported the management outcomes of five cases of limbal dermoid that were managed during infancy and childhood. Oculoauriculovertebral dysplasia or Goldenhar syndrome was present in one of the infants treated. Limbal dermoid, preauricular skin appendages, Duane's disease, tetralogy of Fallot, and sacral agenesis were all present. The limbal dermoid was the only finding in the other patients. Although some limbal dermoid didn't affect the vision, others can affect the vision by inducing astigmatism leading to amblyopia in children. In addition to cosmetic reasons, visually significant limbal dermoid should be surgically excised and followed by aggressive amblyopia treatment. Patching and glasses are the mainstays of treatment. Timing and method of intervention depend on the size and location.

## Introduction

Dermoid cysts are true hamartomas that form when tissue gathers beneath the surface of the skin. Hair, teeth, or nerves may be present in these cysts. They normally show up at the time of birth [1]. Dermoid cysts are most commonly found on the head and neck, although they can also occur in the ovaries, spine, or other parts of the body. Although dermoid cysts are normally innocuous, they are frequently surgically removed [1]. Internal bleeding, infection, and malignancy are just some of the consequences that can emerge from the growth of a dermoid cyst [2].

Epibulbar dermoids involve the globe in children and developed in abnormal locations, which are triggered by a congenital overgrowth of normal tissues by collagenous connective tissue covered by epidermoid epithelium. More than 85% of these lesions are found in the bulbar conjunctiva, limbus, cornea, and/or caruncles, and they can appear unilaterally or bilaterally [3]. The current medical standard of care for grade I pediatric limbal dermoids is to be conservative. Excision, amniotic membrane and limbal stem cell transplantation, and lamellar keratoplasty are recommended in phases II and III. In comparison to standard methods of excision and lamellar keratoplasty, a combination of these approaches appears to produce better and more stable long-term ocular surface cosmesis with fewer complications. To achieve the best results, care of amblyopia must be continued after surgical excision, even if the procedure is performed at a younger age [4]. Multiple preoperative considerations should be considered for patients with limbal dermoid. These include referring the case to a pediatrician who specializes in genetics in order to determine the type of dermoid [5]. Furthermore, brightness (B)-scan imaging is recommended to validate the case presentation, and MRI should be utilized if the lesion has expanded into the conjunctival fornix or lateral canthus [5-7].

## Case presentation

**Figure 1:**
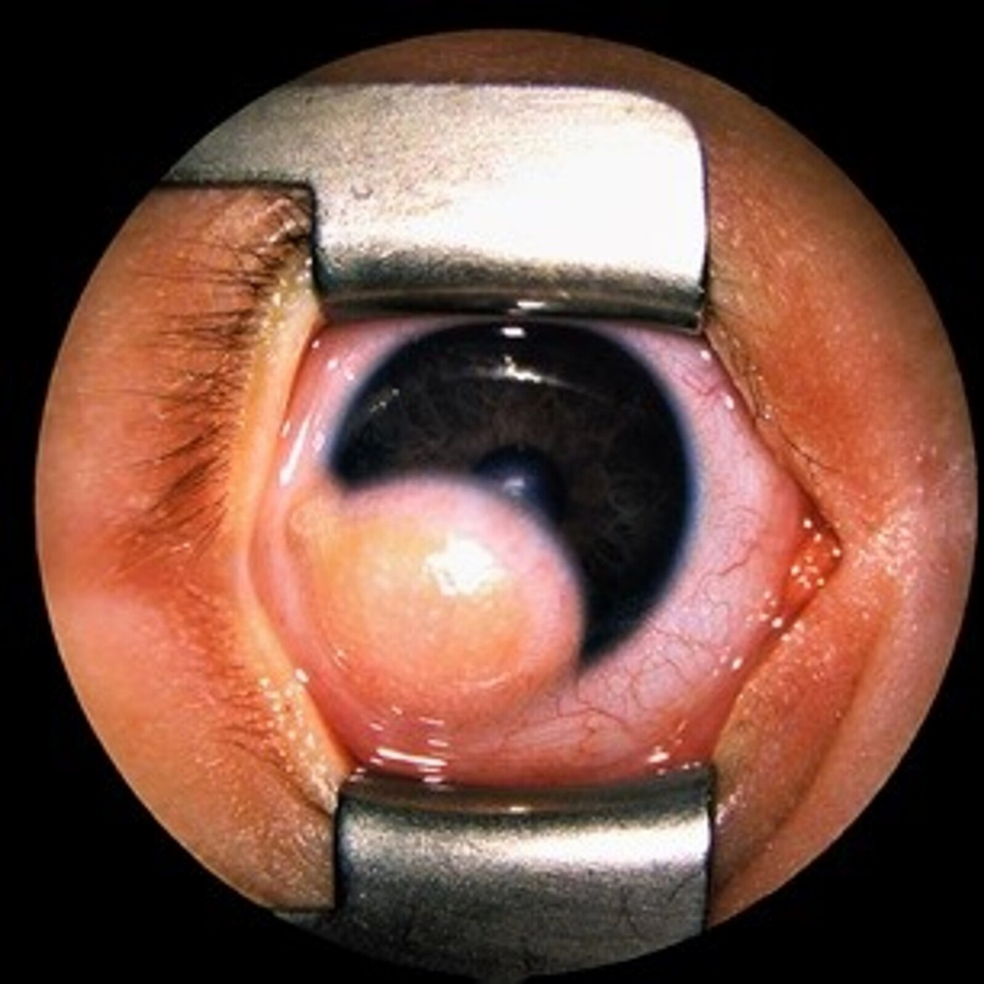
Preoperative clinical presentation of limbal dermoid case (first case)

**Figure 2:**
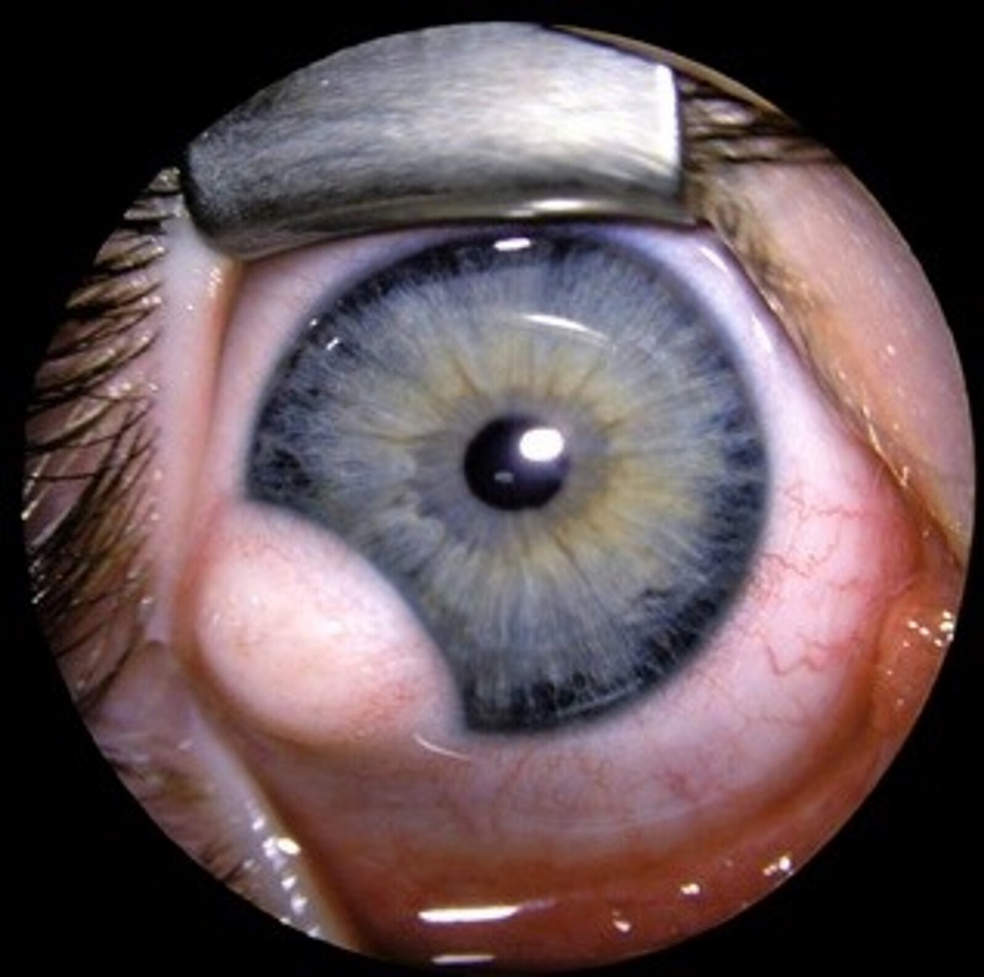
Preoperative clinical presentation of limbal dermoid case (second case)

The focus of this research was to report on the results of five cases with limbal dermoid that were treated during infancy and childhood. The changes in visual acuity, cycloplegic refraction, and astigmatism after therapy were the key outcome measures evaluated. Oculoauriculovertebral dysplasia (OVD) or Goldenhar syndrome was present in one of the infants treated (case number two). Limbal dermoid, preauricular skin appendages, Duane's disease, tetralogy of Fallot, and sacral agenesis were all present. The limbal dermoid was the only finding in the other patients. Figures 1, 2 below presents the preoperative clinical presentation of two limbal dermoid cases.

**Figure 3:**
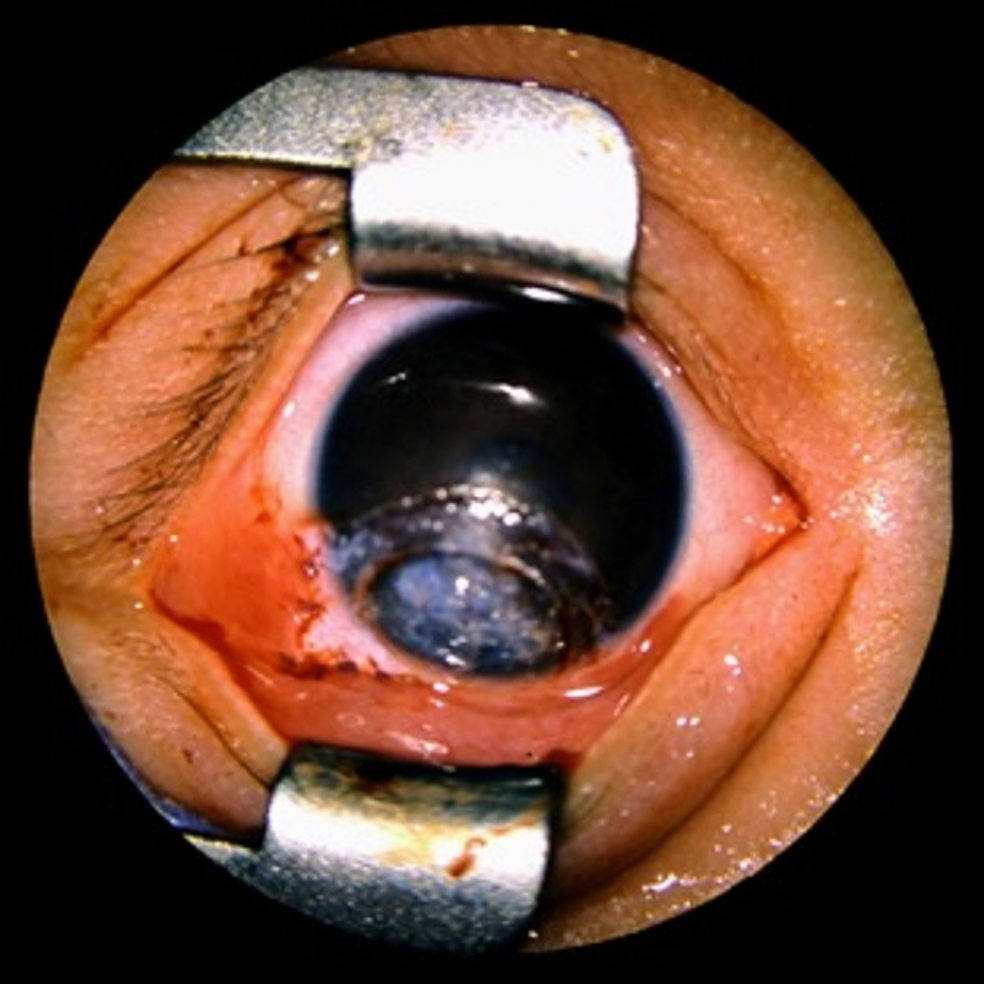
Postoperative clinical presentation of limbal dermoid case (first case).

**Figure 4:**
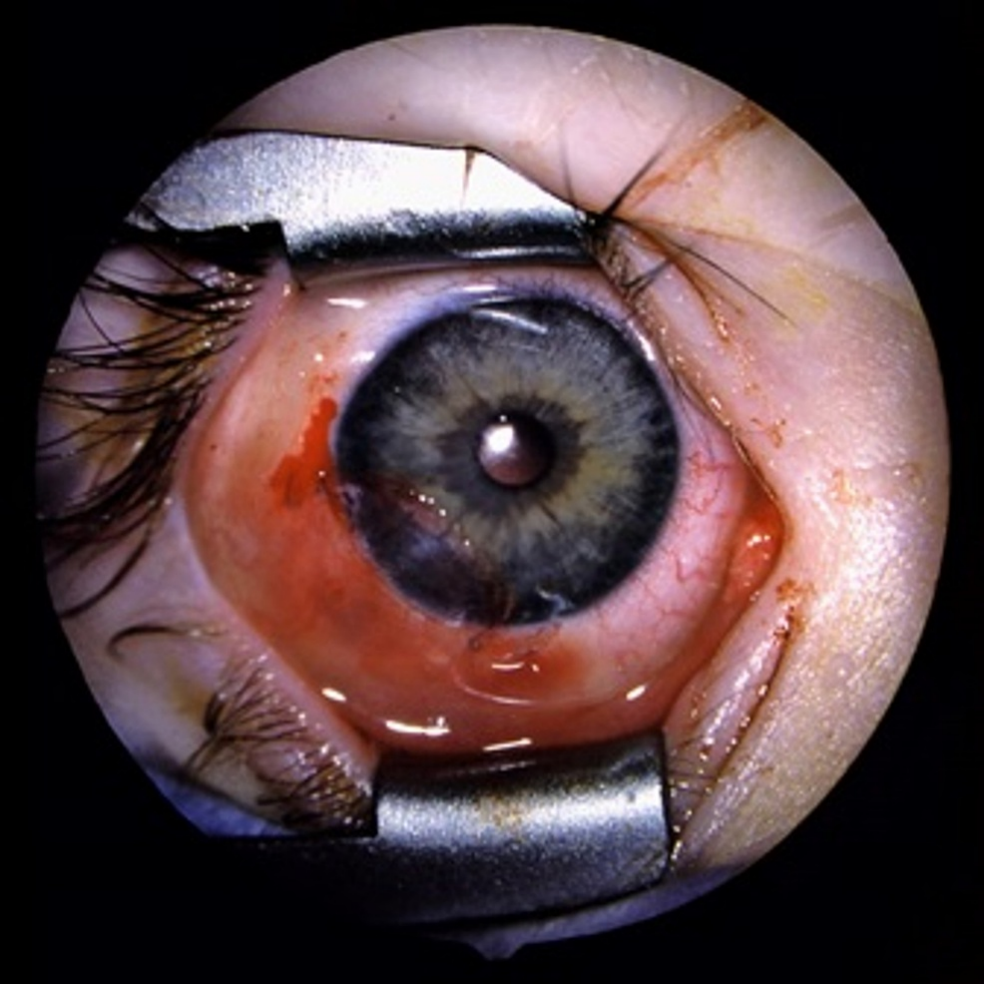
Postoperative clinical presentation of limbal dermoid case (second case).

Figures 3, 4 below shows the postoperative clinical presentation of case one and two.

The limbal dermoid was the only finding in the other patients. An overview of patient characteristics and management outcomes is presented in Table 1.

**Table 1:** Patients' characteristics and management outcomes BCVA: Best-corrected visual acuity; CR: Cycloplegic refraction; OD: Oculus dexter; OS: Oculus sinister; Rt.: Right; Lt.: Left; OU: Oculus uterque; CSM: Central Steady Maintained.

	At presentation										Post-surgery			
Case	Age at presentation	Gender	BCVA	CR	Eye		Size	Age at surgery	Procedure	BCVA		CR	Additional management	
1	18 days	Male	Response to light (OU)	OD: +3.00+3.00 x 180 OS: +3.00	Rt.		8x7 mm	3 weeks	Excision without graft	CSM (OU) at age of 2 and 12 months		At 2 Months: OD: +2.00+4.00 X 150 OS: +1.75	Glasses + patching Lt. eye	
												At 12 Months: OD +2.00+4.50x150 OS+1.50		
2	11 weeks	Male	CSM	OD: 3.00/+2.50 x 120 OS: +5.00	Rt.		8x8 mm	2.5 years	Excision without graft	At 3 years: OD: 20/30 OS: 20/30		At 3 years: OD: +3.50+3.50 x 125 OS: +3.5/+0.50 x 90	Glasses + patching Lt. eye	
										At 5 years: OD: 20/30 OS: 20/30		At 5 years: OD: +3.50+3.50 x 120 OS: +3.00		
										At 8 years: OD: 20/40 OS: 20/25		At 8 years: OD: +2.50+4.25 x 135 OS: +2.50		
3	5 weeks	Male	CSM (OU)	OD: +1.75 OS: +0.50 +2.50 x 90	Lt.		3x4 mm	5 years	Excision without graft	At 5 years: OD: 20/30 OS: 20/50		At 5 years: OD: Plano. OS: -0.25/+3.00 x 90	Glasses + patching Rt. eye	
										At 8 years: OD: 20/20 OS: 20/70		At 8 years: OD: Plano. OS: -0.25/+3.00 x 90		
4	7 months	Male	CSM (OU)	OD: -1.00+1.00x90 OS: -1.50+5.00 x 45	Rt.		6x7 mm	Not done	Not done (observation until excision for cosmetic reason)	CSM (OU) at age of 1.5 years		At 1.5 years OD: -1.00/+1.00 x 90 OS: -1.50/+5.00 x 45	Glasses + patching Rt. eye	
5	13 year	Female	OD: 20/20 OS: 20/20	OD: +0.25/+0.25 x 180 OS: +0.25+0.25x180	Rt		1x2 mm	Not done	Not done (observation until excision for cosmetic reason)	Same BCVA after 6 months and 1 year		Same BCVA after 6 months and 1 year	None	

## Discussion

Grade I dermoids are characterized by having smaller lesions in terms of height and diameter, causing only minor astigmatism of, 1 D with modest surface irregularity, and parents report relatively good compliance with spectacle correction. The literature's principal recommendation is to "leave these lesions alone," [8, 9]. Small asymptomatic grade I limbal dermoids should not be removed since they can induce postoperative pseudopterygium and scarring. These children should be closely observed and inspected in the office on a regular basis, not only to ensure their safety but also to give their parents peace of mind. During each office checkup, which should be done every two to three months, visual acuity, the presence/absence of amblyopia, and advice on occlusion therapy should all be determined [4]. When possible, the size of the lesion should be documented and evaluated using digital photography, stereo acuity, visual acuity, cycloplegic refraction, and gonioscopy [4]. These serial examinations should be continued unless the following criteria for surgical intervention are met: development of clinically significant anisometropia; lack of compliance with either follow-up or spectacle correction; impending or established amblyopia; growth of the limbal dermoid induces marginal dellen, resulting in surface disease and increasing anisometropic astigmatism; and esthetic considerations [4]. There are clinical grounds to proceed with surgical excision and anterior surface reconstruction in patients with a grade I limbal dermoid. If a child or his or her parents refuse to use corrective spectacles, even for minor astigmatism, surgical excision may be considered in the case of amblyopia. If adherence to spectacle wear is good in the setting of big, regular, and oblique astigmatism, and proper follow-up for therapeutic management of amblyopia is possible, surgical surgery may be postponed. If you have amblyopia, you should do everything possible to treat it medically, including wearing glasses and undergoing occlusion therapy [4, 10, 11].

Depending on the degree of the lesion, a range of surgical procedures have been documented in the literature, ranging from simple excision to lamellar and/or penetrating keratoplasty with relaxing corneal incision [12-14]. The depth, size, and location of such lesions are all important considerations. Surgical excision followed by reconstructive sutureless multilayered amniotic membrane transplantation and corneal-limbal scleral donor graft transplantation are two further procedures [15-18]. Due to the clinical morphology of limbal dermoid cases, a biopsy is rarely used, and pathological examination of removed tissue is important after excision [5, 19].

Our findings were in the same line with the results of previous studies [8]. Robb RM conducted a retrospective review of 17 patients to assess the refractive errors associated with limbal dermoids before and after treatment. Only 13 patients (aged between eight months and 15 years) had one or more diopter of astigmatism in the affected eye. The average of pre and postoperative astigmatism was 2.35 and 2.67 diopters, respectively, which reflected a minimal change in the amount of astigmatism [8]. Watts et al. curred a retrospective study on 49 children (51 eyes), aged between one month and 15 years (mean age at surgery was 4.4 +/- 3.8 years), treated for limbal dermoids. From 1900 to 2000, 48 eyes were treated with dermoid excision and lamellar keratoplasty, two eyes were treated with simple excision, and one eye underwent penetrating graft. They recorded preoperative and postoperative refraction in 23 patients only. Of those, ten patients presented with preoperative astigmatism. Following the surgery, four children showed a slight improvement in their astigmatism, three experienced deteriorations of more than one diopter, and three remained the same [8]. In a retrospective Taiwanese study of 10 patients aged 5.7-22.4 years with grade II limbal dermoids who underwent lamellar keratoscleroplasty with full-thickness central corneal grafts, Shen et al. reported that the mean latest postoperative BCVA and earliest BCVA were 6/10 and 6/30, respectively, and the improvement in BCVA after treatment for amblyopia and surgery was 4.9 3.6 lines on the Snellen Patients with preoperative astigmatism of 6.0 D (9.7 1.0 D; n = 4) experienced a substantial reduction in astigmatism of 5.2 1.7 D after surgery. Patients with preoperative astigmatism of 6.0 D (3.4 0.2 D; n = 5) experienced no significant increase in astigmatism after surgery [13]. One patient exhibited substantial corneal opacity after surgery, whereas the other three had a mild bluish scleral tint. Surgical complications included prolonged re-epithelialization, interface neovascularization, graft rejection, and steroid-induced glaucoma [13].

In our study, one child had astigmatism less than one diopter not affecting the vision (case number five), and four had astigmatism greater than one diopter. Astigmatism either increased minimally or remained the same in the treated children. These observations indicate that surgery does not appear to change the associated astigmatism, even if performed at a relatively early age. Similar to the previous studies [8], the improvement of visual acuity in some cases in our study seemed to be related to the use of glasses and patching of the non-involved eye.

## Conclusions

Although some limbal dermoid didn't affect the vision, others can affect the vision by inducing astigmatism leading to amblyopia in children. In addition to cosmetic reasons, visually significant limbal dermoid should be surgically excised and followed by aggressive amblyopia treatment. Patching and glasses are the main stay of treatment. Timing and method of intervention depend on the size and location. Rather than lamellar keratoplasty (deep or superficial), excision of the dermoid from the sclera and partial keratectomy, followed by volumetric filling of the residual corneal defect with fresh multilayered amniotic membrane and pericardial patch graft on the sclera with overlying conjunctival autologous limbal stem cell transplantation may provide the best functional, refractive, and cosmetic results.
